# Staging and monitoring of childhood rhabdomyosarcoma with flow cytometry

**DOI:** 10.3892/ol.2014.1854

**Published:** 2014-02-04

**Authors:** HONGQIANG SHEN, YONGMIN TANG, AO DONG, HUAMEI LI, DIYING SHEN, SHILONG YANG, HONGFENG TANG, WEIZHONG GU, QIANG SHU

**Affiliations:** 1 Division of Hematology and Key Laboratory of Reproductive Genetics, Zhejiang University, Ministry of Education, Hangzhou, Zhejiang 310003, P.R. China; 2 Division of Pathology, Children’s Hospital of Zhejiang University School of Medicine, Hangzhou, Zhejiang 310003, P.R. China; 3 Division of Surgery, Children’s Hospital of Zhejiang University School of Medicine, Hangzhou, Zhejiang 310003, P.R. China

**Keywords:** rhabdomyosarcoma, bone marrow, flow cytometry, minimal residual disease, prognosis

## Abstract

Patients with metastatic rhabdomyosarcoma (RMS) have a poor prognosis. The detection of contaminating RMS cells in the bone marrow (BM) is important in clinical staging and risk assessment. The cytological examination of the BM remains the gold standard for the diagnosis of RMS, but has a limited sensitivity. In the present study, 32 BM and two cerebrospinal fluid (CSF) samples from 11 patients with suspected metastasis were analyzed by flow cytometry (FCM) with ganglioside D2 (GD2) conjugated with fluorescein isothiocyanate, cluster of differentiation (CD)90-phycoerythrin, CD45-peridinin chlorophyll protein and CD56-allophycocyanin monoclonal antibody cocktail in parallel to morphological examination at diagnosis or during treatment. Five samples (14.7%) were positive for RMS onup morphological examination. By FCM, 16 samples (47.1%) were positive for RMS. A significant difference was identified between the two methods. The four-color FCM assay successfully detected RMS cells in BM samples to a level of 0.01% (1 per 10^4^ cells). RMS cells demonstrated a phenotype with CD56^+^/CD90^+^/CD45^−^/GD2^−^ expression, which is different from the CD56^+^/CD90^+^/CD45^−^/GD2^+^ expression phenotype in neuroblastoma cells. The follow-up of four patients by FCM demonstrated that two patients became minimal residual disease-negative following two and four cycles of chemotherapy, respectively, and survived. The other two cases remained FCM-positive despite receiving four courses of chemotherapy and consequently succumbed to progressive disease. In addition, FCM analysis of the CSF samples from one patient confirmed a diagnosis of CSF metastasis with RMS. In conclusion, FCM may have a role not only in staging and monitoring the effects of therapy, but also in providing diagnostic confirmation of CSF metastasis with RMS.

## Introduction

Rhabdomyosarcoma (RMS), a form of soft-tissue sarcoma, is the most common type of extracranial neoplasm in childhood and represents ~4.5% of childhood cancer (1). RMS shows a wide range of biological, genetic and morphological characteristics and exhibits a diverse clinical behavior. On the basis of histopathological criteria, RMS in childhood is classified into the two main subtypes of embryonal RMS (eRMS; 80%) and alveolar RMS (aRMS; 20%). Patients that present with a localized tumor have a good prognosis. However, ~15% of patients with RMS have high-risk stage IV diseases with bone marrow (BM) involvement and a poor clinical outcome, with a three-year survival rate of ~15% (1,2). The use of myeloablative chemotherapy in combination with autologous BM or peripheral blood stem cell transplantation rescue may be required to treat children with stage IV disease (3,4). Thus, the detection of BM involvement at diagnosis is critical for accurate staging and risk assessment. In addition, minimal residual RMS cell assays may be used during therapy to assess the early response to treatment and predict relapse. Morphological screening of BM aspirates has been the gold standard for a number of years. Considering a detection sensitivity level of 1% tumor cells for cytological screening of BM samples, it appears conceivable that morphological techniques alone lack the sensitivity to monitor minimal residual disease (MRD). For this reason, new methods have been developed. Detection of specific transcripts, such as PAX3/7-FKHR, in RMS patients by reverse transcription-polymerase chain reaction (PCR) has been previously demonstrated (5–7). Although PCR amplification has a high sensitivity, it is relatively complicated and time consuming in application. It has also been shown that almost all eRMS and ~25% of aRMS are translocation-negative, and PCR methods may not be used for BM metastasis detection in such patients (5).

Flow cytometry (FCM) is widely used for the diagnosis of leukemia and lymphoma, which has been previously shown to increase the overall diagnostic accuracy in a number of studies. FCM has a high sensitivity and is also commonly used to assess MRD in leukemia. To date, few studies have been reported that evaluate the utility of FCM immunophenotyping for the diagnosis of BM metastasis in patients with RMS. Additionally, detection by FCM of minimal residual RMS cells in the BM during therapy to enable an evaluation of the efficacy of therapy has not been reported. Previous studies have shown that RMS cells typically exhibit a cluster of differentiation (CD)45^−^/CD56^+^/CD90^+^/myogenin^+^ phenotype with variable expression of CD57, desmin, vimentin and CD99 in fine-needle aspiration cytological specimens (8). Bozzi *et al* (9) previously reported that tricolor FCM detection of the CD56^+^/CD90^+^/CD45^−^ immunophenotype is extremely useful for the diagnosis of BM metastasis in patients with RMS, but the efficacy of FCM in monitoring therapeutic effects during and/or after chemotherapy has not been previously evaluated. In addition, neuroblastoma (NB) is the most common type of non-hematopoietic metastatic tumor in children and also has a CD56^+^/CD90^+^/CD45^−^ immunophenotype (9–11). The formation of a differential diagnosis of RMS from NB using this immunophenotype is difficult with FCM. Previous studies have shown that ganglioside D2 (GD2) is expressed by neuroectodermally-derived tumors, such as NB and retinoblastoma (11–14). RMS cells are negative for GD2 (9,10). In the current study, a four-color FCM assay was developed with a CD56/CD90/CD45/GD2 monoclonal antibody cocktail to stage RMS and detect MRD in BM samples during and/or after chemotherapy, to evaluate therapeutic efficacy.

## Materials and methods

### Patients and samples

Between November 2008 and December 2012, 27 cases of children with RMS were diagnosed by histopathology at the Children’s Hospital of Zhejiang University School of Medicine (Hangzhou, China). The patients consisted of 13 females and 14 males, with a median age of six years (range, 1–14 years). The primary sites included the genitourinary tract (n=8), the head and neck (n=7), the trunk and extremities (n=5), the post-peritoneum (n=4) and the pelvis (n=3). In total, 25 cases presented as the embryonal type and two as the alveolar type in histopathology. In addition, 32 BM and two cerebrospinal fluid (CSF) samples were obtained from 11 patients with suspected metastasis and analyzed by FCM in parallel to conventional diagnostic procedures at the time of diagnosis or during treatment. The study was approved by the local ethics committee and written informed consent was obtained from the parents or guardians of each patient in accordance with the Helsinki protocol. The treatment of RMS included multimodal therapy combined with surgery (complete primary tumor excision and lymph node removal), chemotherapy [vincristine (1.5 mg/m^2^ ), actinomycin D (12 μg/kg) and cyclophosphamide (VAC; 300 mg/m^2^)] and radiation (40–50 Gy at 1.5–1.8 Gy/fraction) based on the Children’s Oncology Group (COG) protocol. Chemotherapy was performed monthly.

### Control group

BM samples were obtained from nine cases of clinically or pathologically diagnosed non-neoplastic disease and an additional 27 cases diagnosed with other types of clinical neoplastic diseases, consisting of 12 cases of acute lymphocytic leukemia and 15 cases of NB.

### Morphological and immunochemical evaluation

The aspirates of BM were smeared onto at least three slides and then stained and evaluated using the May-Grünwald-Giemsa (MGG) procedure. The CSF samples were centrifuged at 800 × g for 8 min. The supernatant was removed and the cell pellets were smeared onto the three slides. Following air-drying, the slides were stained with MCG and immunochemical stains. Immunoreactivities to myogenic differentiation 1 (MyoD1) and desmin in BM biopsied samples were used as specific RMS markers. Antibodies for MyoD1 and desmin (Mouse anti-human MyoD1 and desmin monoclonal, respectively) were obtained from DakoCytomation (Glostrup, Denmark). Samples were stained immunochemically using the ChemMate™ Dako EnVision™ two-step system (horseradish peroxidase; DakoCytomation). Appropriate positive and negative controls were set. Microscopic examinations were performed by at least two experienced pathologists.

### Four-color FCM assay

The BM samples were adjusted to 1×10^7^ mononuclear cells per ml. The cell pellets of CSF were resuspended with 100 μl phosphate-buffered saline. Cell suspensions (100 μl) were stained with GD2 conjugated with fluorescein isothiocyanate (FITC) (supplied by Professor C. Patrick Reynolds of the Children’s Hospital Los Angeles, Los Angeles, CA, USA), CD90-phycoerythrin (PE), CD45-peridinin chlorophyll protein (PerCP) and CD56-allophycocyanin (APC) (Becton-Dickinson, Franklin Lakes, NJ, USA) for 30 min at 4°C in the dark. The background of non-specific antibody uptake was evaluated by staining in parallel with isotype-matched immunoglobulin (Ig)G2a-FITC, IgG1a-PE, CD45-PerCP and CD56-APC (Becton-Dickinson). At least 50,000 events were acquired and analyzed using the CellQuest (version 3.2) software of the fluorescence-activated cell sorting calibur flow cytometer (Becton-Dickinson) and FlowJo7.6 software (Tree star, Inc., Ashland, OR, USA), respectively.

The sensitivity of the four-color FCM assays was evaluated using spiking experiments. The absolute RMS cells were calculated in a BM sample from a patient with BM involvement by providing the total white blood cell count and the percentage of RMS cells from a complete/differential cell count. Next, the RMS cells were diluted with the normal hematopoietic cells from the BM at the following indicated proportions: 1, 0.1, 0.01 and 0.001%. The dilutions from 1 to 0.001% were evaluated by FCM. FCM results (positive or negative for malignancy) were compared with those from BM and CSF cytology, and the diagnosis was established by the clinicians.

### Statistical analysis

Data are presented as the median when continuous and as the absolute and relative frequency when categorical. The diagnostic specificity of FCM was calculated by the χ^2^ test. McNemar’s test was used to evaluate the differences between FCM and cytological study for the BM involvement of the tumor cells. All statistical analyses were performed using the Statistical Package for the Social Sciences (SPSS) software, version 12.0 (SPSS, Inc., Chicago, IL, USA). P<0.05 was considered to indicate a statistically significant difference.

## Results

### Morphological and immunohistochemical observations

**Figure 1:**
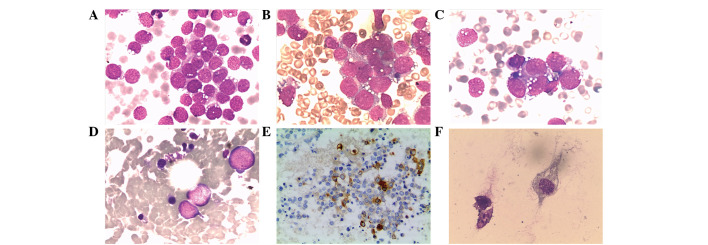
Morphological and immunophenotypical features of RMS. (A–C) BM smears of patients 1, 2 and 3, respectively, with extensive infiltration by a homogeneous population of the primitive malignant cells. The cells are gathered in small clusters (MCG stain; high-power view). (D) BM smears of patient 4 in which the scattered malignant cells were found with a retrospective morphological examination under the implication of flow cytometry (FCM) positivity (MCG stain; high-power view). (E) RMS cells positive for desmin in BM biopsy (hematoxylin and eosin stain; magnification, ×200). (F) RMS cells in cerebrospinal fluid (CSF) specimens showing degenerative changes (MCG stain; high power view). RMS, rhabdomyosarcoma; BM, bone marrow; MCG, May-Grünwald-Giemsa.

A total of 11 cases with suspected metastasis at diagnosis underwent BM examination. Smears from three cases showed the feature of BM metastasis by RMS, and these patients were determined as stage IV RMS based on the Intergroup RMS Study Group (IRSG). All cases presented as the embryonal type. Clinical characteristics in cases with BM metastasis are shown in Table I. The neoplastic cells in the BM from these three cases demonstrated a similar morphology. Smear preparations showed that a homogeneous population of the primitive malignant cells gathered in small clusters (Fig. 1A–C). The cells demonstrated cytoplasmic immunoreactivity with desmin, confirming the diagnosis of RMS (Fig. 1E).

The treatment to RMS included multimodal therapy with a combination of surgery, chemotherapy and radiation based on the COG protocol. The three patients with BM involvement received chemotherapy with the VAC regimen for 8–12 months following surgery. The follow-up with morphological examination demonstrated that RMS cells had not been found in the BM following the first cycle of chemotherapy in all patients. One patient exhibited repeated twitching and altered consciousness following 10 cycles of chemotherapy. A lumbar puncture was performed when the patient was admitted and a routine analysis of CSF showed a cell count of 90×10^6^/l with 90% mononuclear cells. A smear examination of the pellet of CFS showed a number of neoplastic cells with vesicular nuclei and abundant densely eosinophilic scattered cytoplasm (Fig. 1F). The cells were positive for desmin that was compatible with RMS.

### FCM immunophenotyping observations

**Figure 2:**
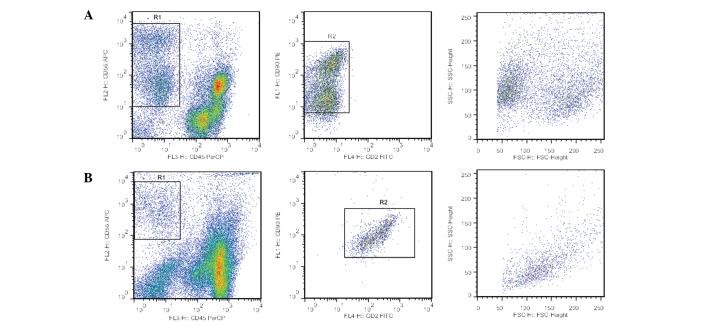
Four-color FCM analysis of bone marrow specimens from patients with RMS and NB using GD2-FITC/CD90-PE/CD45-PerCP/CD56-APC monoclonal antibody combination. (A) RMS cells demonstrating an immunophenotype with CD56^+^/CD90^+^/CD45^−^/GD2^−^ expression. (B) NB cells demonstrating an immunophenotype with CD56^+^/CD90^+^/CD45^−^/GD2^+^ expression (R2 gate refers to R1). RMS, rhabdomyosarcoma; NB, neuroblastoma; RMS, rhabdomyosarcoma; FITC, fluorescein isothiocyanate; PE, phycoerythrin; PerCP, peridinin chlorophyll protein; APC, allophycocyanin; CD, cluster of differentiation; FCM, flow cytometry.

Four out of the 11 BM samples obtained from 11 patients were positive for RMS cells by FCM at diagnosis, from which, three cases were in accordance with the results of the morphological evaluation. RMS cells demonstrated an immunophenotype with positive CD90 and CD56 expression, but lacking expression of CD45 and GD2 antigen (Fig. 2A). The percentage of positive cells was 29.3, 12.3, 6.8 and 0.35% among the total nucleated cells in these four cases, respectively. FCM showed an extremely low percentage (0.35%) of RMS cells in the morphologically-negative case. A retrospective morphological examination of the BM smear from this case was carefully performed due to its FCM positivity, and the results showed neoplastic cells scattered in the background (Fig. 1D). This patient, previously stage II, had their staging diagnosis modified to stage IV RMS by their clinician and were administered stage IV treatment regimens. A total of 15 cases of metastatic NB diagnosed in the BM specimens were examined by FCM. The FCM results showed that the NB cells demonstrated a CD56^+/^CD90^+^/CD45^−^/GD2^+^ expression immunophenotype (Fig. 2B). The analysis of the 21 BM samples obtained from nine patients with non-neoplastic diseases and 12 patients with acute lymphoblastic leukemia revealed no cells with the CD56^+^/CD90^+^/CD45^−^/GD2^−^ or CD56^+^/CD90^+^/CD45^−^/GD2^+^ phenotypes.

**Figure 3:**
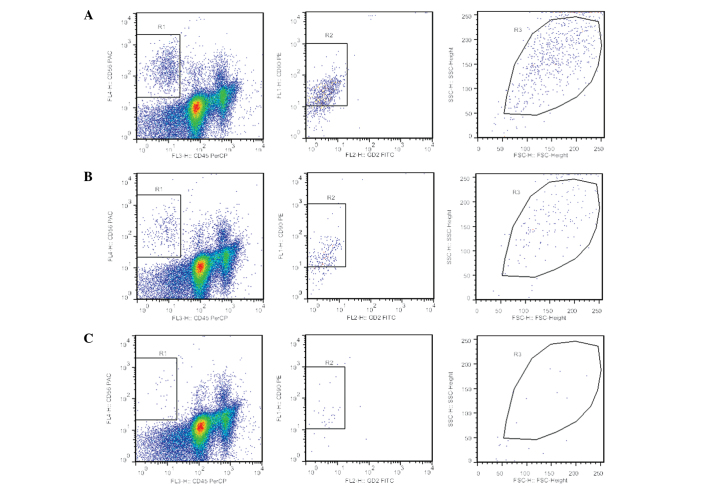
Sensitivity of FCM analysis of RMS cells. RMS cells were diluted with the normal hematopoietic cells from bone marrow (BM) at the following indicated proportions: (A) 1%, (B) 0.1% and (C) 0.01%, and stained with GD2-FITC/CD90-PE/CD45-PerCP/CD56-APC monoclonal antibody. FCM had a sensitivity of 0.01% based on the spiking experiment. RMS, rhabdomyosarcoma; FITC, fluorescein isothiocyanate; PE, phycoerythrin; PerCP, peridinin chlorophyll protein; APC, allophycocyanin; CD, cluster of differentiation; FCM, flow cytometry.

To evaluate the therapy response during treatment and in order to predict a prognosis, RMS-MRD detection by FCM with GD2-FITC/CD90-PE/CD45-PerCP/CD56-APC was established. The sensitivity of the FCM assays was evaluated using spiking experiments. The results showed that when 5×10^4^ cells were evaluated, one RMS cell in every 10^4^ normal mononuclear cells (0.01%) was detected (Fig. 3).

**Figure 4:**
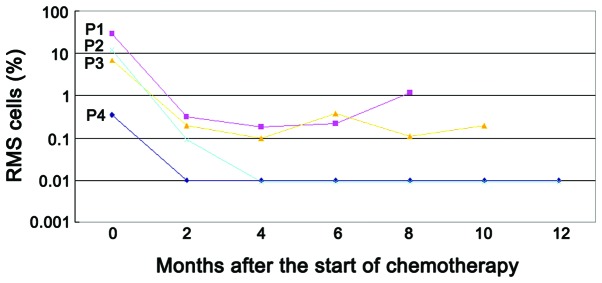
Detection of MRD by flow cytometry (FCM) using a GD2-FITC, CD90-PE, CD45-PerCP and CD56-APC monoclonal antibody combination in four patients with RMS. The cells with the phenotype of >0.01% CD56^+^/CD90^+^/CD45^−^/GD2^−^ expression in the bone marrow (BM) sample were considered positive. Two patients (P2 and P4) became MRD-negative following two and four cycles of chemotherapy and remained alive. The other two cases (P1 and P3) maintained their positivity despite receiving four courses of chemotherapy and succumbed to progressive disease at 8 and 12 months, resepctively, following the initiation of chemotherapy. RMS, rhabdomyosarcoma; MRD, minimal residual disease; P, patient; FITC, fluorescein isothiocyanate; PE, phycoerythrin; PerCP, peridinin chlorophyll protein; APC, allophycocyanin; CD, cluster of differentiation.

**Figure 5:**
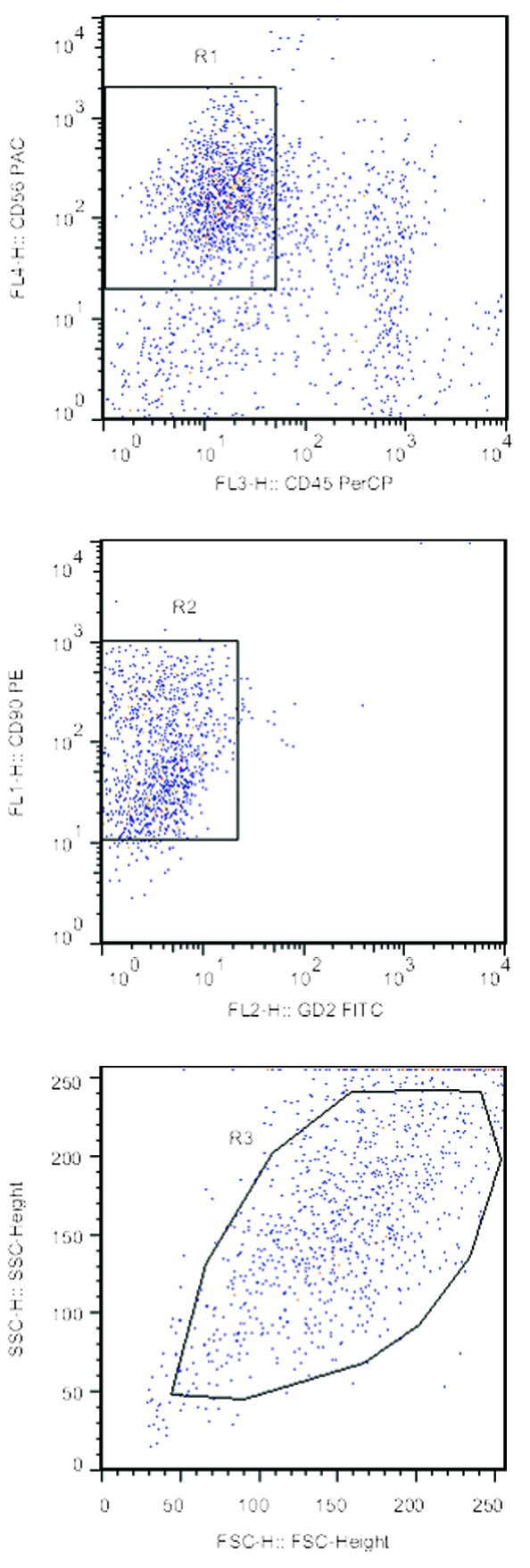
Four-color FCM analysis of a cerebrospinal fluid (CSF) specimen from the patient with rhabdomyosarcoma (RMS). FCM demonstrating a population of cells with the CD56^+^/CD90^+^/CD45^−^/GD2^−^ phenotype (R2 gate refers to R1 and R3 refers to R2). FITC, fluorescein isothiocyanate; PE, phycoerythrin; PerCP, peridinin chlorophyll protein; APC, allophycocyanin; CD, cluster of differentiation; FCM, flow cytometry.

In four cases with positive BM at diagnosis, further BM aspirates were obtained during treatment to detect MRD by FCM at the interval of two courses of chemotherapy in order to evaluate the efficacy of the therapy. Follow-up with FCM assays demonstrated notable results (Fig. 4). In total, two patients [patient (P)2 and P4] became MRD-negative in BM, as determined by FCM following two and four cycles of chemotherapy, and remained alive without evidence of disease with follow-up periods of 38 and 49 months, respectively. The other two cases (P1 and P3) remained RMS MRD-positive despite receiving four courses of chemotherapy with 8 and 12 months of follow-up, respectively. Among them, one case (P1) ultimately experienced disease recurrence and succumbed to uncontrolled disease progression eight months after chemotherapy. The other case (P3) exhibited CSF metastasis of RMS following 10 courses of chemotherapy, and continuous neoplastic cells with the CD56^+^/CD90^+^/CD45^−^/GD2^−^ phenotype remaining were observed in CSFs by FCM during treatment (Fig. 5). The patient became unconscious and subsequently succumbed to progressive disease following two cycles of intrathecal chemotherapy.

Five samples (14.7%) were positive for RMS onup morphological examination. By FCM, 16 samples (47.1%) were positive for RMS. A significant difference was identified between the two methods (χ^2^=9.09; P<0.05). The specificity of FCM for diagnosing RMS in BM and CSF samples was 100%.

## Discussion

A proportion of children with RMS present with disseminated disease, including BM involvement. In the present study, 14.8% (4/27) of patients with RMS exhibited high-risk stage IV diseases with BM involvement, similar to the results (15%) previously reported by Dasgupta and Rodeberg (1). The detection of contaminating RMS cells in the BM is important in clinical staging and risk assessment. Cytological examination of the BM remains the gold standard for RMS diagnosis, but has a limited sensitivity. Furthermore, several studies have previously shown that RMS presents with extensive BM involvement and mimics acute leukemia in cytology (15,16). For several decades, FCM immunophenotyping has been confirmed to be essential for the rapid diagnosis, classification and monitoring of therapy in the majority of hematological malignancies, including pediatric leukemias and lymphomas. Conversely, it is rarely used to identify metastatic cells of solid tumors in BM. An important exception is represented by NB stage IV patients, in whom FCM has been used to detect disease in BM during diagnostic or staging procedures (17–19). However, few previous studies have performed an FCM analysis of RMS, and the efficacy of FCM in monitoring therapeutic effects and progress by detecting residual disease following chemotherapy has not been evaluated.

The present study developed a four-color FCM assay using GD2, CD90, CD45 and CD56 to detect RMS cells in BM. In total, three RMS IRSG IV BM samples that had been determined to be infiltrated by neoplastic cells using standard morphology, exhibited the CD56^+^/CD90^+^/CD45^−^/GD2^−^ phenotype. Cases were encountered where the aspirate samples were positive by FCM while negative by morphology analysis. FCM demonstrated the presence of an aberrant population of cells with the CD56^+^/CD90^+^/CD45^−^/GD2^+^ phenotype, which accounted for 0.35% of the cells of the case. This case, previously stage II, was modified to stage IV RMS by a clinician and was administered stage IV treatment regimens and obtained a good clinical outcome. This discrepancy between the cytological and FCM results may be explained by the difference in the sensitivity of the methods used. In the present study, the FCM assay had a sensitivity of 0.01% based on spiking experiments. In general, the detection of malignant cells using a conventional cytology method requires that a minimum of 5% of the neoplastic cells in a sample are identified. The results of the current study indicated that an FCM assay in RMS may identify a group of patients who are at a high risk despite their cytomorphologically-negative BM.

Follow-up examinations using FCM assays demonstrated that two out of the four cytomorphically-negative cases remained FCM-positive following four courses of chemotherapy. The patients clinical outcomes were poor while the clinical outcomes for the other two FCM-negative cases were good. Detection of MRD using FCM may have prognostic or therapeutic implications in advanced RMS. Further studies with follow-up for a larger number of cases are required to validate the prognostic significance of MRD assessment by FCM in children with RMS.

Notably, in the current study, FCM results showed that the cells in the CSF from one patient demonstrated the CD56^+^/CD90^+^/CD45^−^/GD2^−^ expression immunophenotype, indicating the presence of CSF metastasis of RMS. This patient with a primary tumor site in the orbit may have developed metastatic spread into the meninges. A complete metastatic evaluation in orbital sarcoma patients currently includes a lumbar puncture for CSF cytological analysis (20). To date, few studies have analyzed the application of FCM to the study of the diagnosis of CSF metastasis in patients with RMS. Although a cytomorphological examination of the CSF may allow an easy and quick identification of RMS, a paucity of cells in the CSF and the presence of degenerative changes in effusions may result in difficulty in the specific categorization of neoplastic cells (21). Therefore, FCM analysis is extremely useful for the diagnosis of CSF metastasis in patients with RMS, which may also be useful during the follow-up of patients with a high risk of metastasis.

NB is the most common type of extracranial malignant solid tumor in children, accounting for 7–10% of all childhood cancers. In total, ~60% of children and 80% of infants with NB are stage IV at the time of diagnosis with BM or bone metastases (13). Due to the relatively high frequency of metastatic BM involvement in NB, the differential diagnosis of RMS from NB is essential in clinical practice. NB and RMS exhibit a similar morphology, characterized by small, round, relatively undifferentiated cells that may often be morphologically confused. NB and RMS exhibit a CD45^−^/CD56^+^/CD90^+^ phenotype. Previous studies have shown that GD2 is positively expressed by neuroectodermally-derived tumors, such as NB, while it is negative in RMS cells. GD2 may be a useful marker to differentiate between NB and RMS. The results of the current study demonstrated that the four-color FCM assay with the CD45/CD56/CD90/GD2 antibody cocktail is useful in establishing a differential diagnosis between these two malignancies. In the current study, none of the BM samples from patients with non-neoplastic diseases were misdiagnosed as cancer by FCM.

In conclusion, FCM may have a role not only in staging and monitoring the effects of therapy, but also in providing diagnostic confirmation of CSF metastasis in RMS. This technique is simple, quick and cost effective and may be translated to routine practice.

## Figures and Tables

**Table I:** Clinical characteristics of four cases of RMS with BM metastasis.

Patient no.	Gender	Age, years	Primary tumor sites	Metastatic sites	Histological subtype	Stage	Therapy	Chemotherapy	Survival period, months	Follow-up
1	M	8	Head	BM	Embryonal	IV	S+R+C	VAC	8	Succumbed to recurrence
2	M	5	Neck	BM	Embryonal	IV	S+R+C	VAC	38	Alive
3	M	13	Head (orbit)	BM	Embryonal	IV	S+R+C	VAC	12	Succumbed to disease
4	F	10	Post-peritoneum	BM^a^	Embryonal	IV	S+R+C	VAC	49	Alive

a Diagnosed by flow cytometry (FCM).

S, surgery; R, radiation; C, chemotherapy; VAC, vincristine, actinomycin D and cyclophosphamide; M, male, F, female; BM, bone marrow; RMS, rhabdomyosarcoma.
